# Super-Resolution Imaging With Lanthanide Luminescent Nanocrystals: Progress and Prospect

**DOI:** 10.3389/fbioe.2021.692075

**Published:** 2021-09-30

**Authors:** Hongxin Zhang, Mengyao Zhao, István M. Ábrahám, Fan Zhang

**Affiliations:** ^1^ Department of Chemistry, Shanghai Key Laboratory of Molecular Catalysis and Innovative Materials, State Key Laboratory of Molecular Engineering of Polymers, iChem, Fudan University, Shanghai, China; ^2^ Molecular Neuroendocrinology Research Group, Institute of Physiology, Medical School, Centre for Neuroscience, Szentágothai Research Institute, University of Pécs, Pécs, Hungary

**Keywords:** super-resolution imaging, lanthanide-doped nanocrystals, stimulated emission depletion nanoscopy, fluorescence emission difference nanoscopy, *in vivo* imaging

## Abstract

Stimulated emission depletion (STED) nanoscopy has overcome a serious diffraction barrier on the optical resolution and facilitated new discoveries on detailed nanostructures in cell biology. Traditional fluorescence probes employed in the super-resolution imaging approach include organic dyes and fluorescent proteins. However, some limitations of these probes, such as photobleaching, short emission wavelengths, and high saturation intensity, still hamper the promotion of optical resolution and bio-applications. Recently, lanthanide luminescent probes with unique optical properties of non-photobleaching and sharp emissions have been applied in super-resolution imaging. In this mini-review, we will introduce several different mechanisms for lanthanide ions to achieve super-resolution imaging based on an STED-like setup. Then, several lanthanide ions used in super-resolution imaging will be described in detail and discussed. Last but not least, we will emphasize the future challenges and outlooks in hope of advancing the next-generation lanthanide fluorescent probes for super-resolution optical imaging.

## Introduction

**FIGURE 1:**
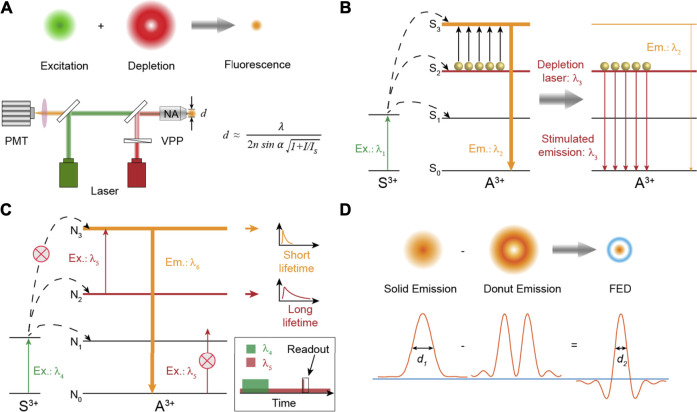
**(A)** Schematic diagram showing the principle of super-resolution imaging and the simplified setup of STED nanoscopy. VPP: vortex phase plate. The formula describes the resolution of STED nanoscopy. *d* is the diameter of the spot that fluoresces. **(B)** The energy transfer pathway of typical Tm^3+^-based nancrystals for super-resolution. Stimulated emission is triggered under a *λ*_3_ laser excitation to deplete the population of energy state S_3_. **(C)** The energy transfer pathway of the excited state absorption process to achieve super-resolution through separated time–domain detection. **(D)** Theoretical results of point spread functions obtained in fluorescence emission difference microscopy. The negative values caused by the intensity subtraction are set to zero to ensure a better spatial resolution.

It is often said that seeing is believing. This applies not only to our daily lives but certainly also to the academic research. For centuries, fluorescence microscopy has greatly facilitated our understanding on the spatial organization and interactions of the biological system (Misgeld et al., 2007; Klar et al., 2000). The main indicator to evaluate the performance of optical micro-imaging is the spatial resolution (Hell and Wichmann, 1994; Hell, 2004). However, as we learned in high school, the resolution of a visible light microscope is usually larger than 200 nm due to the diffraction limit theoretically proposed by Ernst Abbe in 1873, making many nanostructures, such as neurons and cytoskeletons in cells, unresolvable (Betzig et al., 2006). Facing the seemingly insurmountable challenge, several advanced and ingenious methods to break through the bottleneck of the optical resolution in microscopy have been invented so far (Gwosch et al., 2020; Berning et al., 2012; Rust et al., 2006; Sharonov and Hochstrasser, 2006; Bates et al., 2008), one of which is named as stimulated emission depletion (STED) microscopy honored by the 2014 Nobel Prize in Chemistry (Hell, 2015; Sahl et al., 2017). In this design (Figure 1A), two excitation laser beams are required: one laser beam with Gaussian intensity distribution is used to excite probes to generate fluorescence. Another donut laser beam, called the depletion laser, is employed to erase fluorescence signals around the focal spot through stimulated emission so as to reduce the size of the region that fluoresces. Then scanning the little light spot across samples will record a super-resolution image (Harke et al., 2008; Eggeling et al., 2009).

Up until now, the super-resolution imaging of neurons, mitochondria, and cytoskeletons in live cells and live bodies has been realized by STED technology (Eggeling et al., 2009; Sahl et al., 2017; Carola et al., 2018; Masch et al., 2018; Rifka et al., 2018; Grimm et al., 2019; Stephan et al., 2019), in which organic dyes and fluorescent proteins (ATTOs, DAEs, 61*CP*, and so on) that have the fundamental transition property of stimulated emission are commonly used as fluorescent probes (Kolmakov et al., 2015; Butkevich et al., 2018; Uno et al., 2019). For biological samples, a feature resolution of 20 nm could be obtained by STED super-resolution imaging (Sahl et al., 2010). Despite great efforts, some annoying restrictions related to the current fluorescent probes still exist and hamper the improvement of spatial resolution of STED technology. Photobleaching or photostability of the most organic probes due to high power intensity (1–200 MW/cm^2^) of the depletion laser for the desired spatial resolution is a platitude but disturbing problem.^7^ In addition, the high excitation power density of the depletion laser could also cause overheating and phototoxic effects on biological specimens (Waldchen et al., 2015; Masch et al., 2018). Besides, the imaging depth of STED technology is often limited by the short excitation, depletion, and emission (<800 nm) wavelengths because of their strong photo scattering in bio-tissues (Kolmakov et al., 2015). In contrast to conventional biomarkers, quantum dots (QDs) with excellent photostability can also be utilized as standard fluorescent probes in STED microscopy with a resolution of 50 nm (Hanne et al., 2015). However, the excitation power density and excitation wavelengths in these reports still need to be optimized.

Besides the abovementioned fluorescent probes, lanthanide-based luminescent biological probes with near-infrared emissions have been proven to be superior for *in vivo* optical imaging and detection due to their unique optical properties, including non-photobleaching, sharp emissions, and large Stokes shifts (Wang et al., 2014; Zhang et al., 2019; Fan and Zhang, 2019; Zhong et al., 2019). So far, researchers have demonstrated the potential applications of several kinds of lanthanide-doped nanomaterials on super-resolution optical microscopy (Liu et al., 2017; Zhan et al., 2017; Huang et al., 2018; Krause et al., 2019; Dong et al., 2020). In review of the already reported results, here we first describe several mechanisms for super-resolution optical microscopy based on lanthanide nanostructures and a STED-like setup. Then, we present and analyze the development and applications of these inorganic fluorescent probes for optical micro-imaging. Last, the emphasis of the review is placed on the challenges and opportunities in advancing next-generation fluorescent probes for super-resolution optical imaging.

## Designing a Scheme for Breaking Through the Diffraction Limit

Taking advantage of the STED setup, three disparate mechanisms are proposed to obtain a high spatial resolution of optical imaging. They are stimulated emission depletion fluorescence, excited state absorption, and fluorescence emission difference, all of which rely on emitting transition (switching) between two states, typically a fluorescent “on” state and a dark, nonfluorescent “off” state, allowing the limiting character of diffraction to be suppressed with or without data processing.

### Stimulated Emission Depletion Fluorescence

Stimulated emission to deplete fluorescence for super-resolution imaging, experimentally realized by Stefan W. Hell in 1999 (Klar and Hell, 1999), is an efficient and widely used method now. For organic dyes, stimulated emission from a suitable laser excitation is a fundamental property of electron transition. However, due to the abundant energy levels of trivalent lanthanide ions, excitation under two-color fields will always cause enhanced luminescence intensity of lanthanide activators through excited state absorption (Zhou et al., 2013a; Chen et al., 2015; Zhang et al., 2016). Until the seminal paper published in 2017, Jin et al. reported that the conventional stimulated emission of Yb^3+^/Tm^3+^ co-doped nanocrystals could be triggered by an 808-nm laser illumination due to the population inversion of a metastable ^3^H_4_ level caused by intense cross-relaxation (CR) at high concentrations of Tm^3+^ ions (Liu et al., 2017). The stimulated emission mechanism can be simplified as shown in Figure 1B. Electrons on the energy level S_2_ will be populated to the S_3_ state to emit fluorescence with the wavelength at *λ*_2_ when the lanthanide materials are only excited at laser *λ*_1_. After the depletion laser *λ*_3_, matching the emission band of the S_2_ → S_0_ transition, is turned on simultaneously, electrons on the S_2_ state will be discharged to the ground state S_0_ through a stimulated emission process, which in turn greatly reduces the electron population of the energy level S_3_ and thus exhausts its fluorescence intensity. It should be noted that one crucial prerequisite is the population inversion of the S_2_ state relative to the S_0_ ground level to maximally alleviate the ground state absorption of S_0_ on the depletion laser *λ*_3_. In STED nanoscopy, the resolution obeys the formula (Wildanger et al., 2009):

dSTED≈ λNA1+IIS (1)

where λ denotes the excitation wavelength of the depletion laser and NA represents the numerical aperture of the objective lens. I is the maximum intensity of the depletion laser and $I_{S}$ , termed saturation intensity, is defined as the power density point to obtain the half value of the maximum fluorescence intensity.

### Excited State Absorption

Benefiting from the abundant energy states of trivalent lanthanide ions, diversified excitations on lanthanide-doped materials can provide a desired luminescent behavior. It was reported that depleted fluorescence under two laser co-excitation could be realized through excited state absorption (ESA) as well (Kolesov et al., 2011; Wu et al., 2015). The schematic energy transfer pathway is depicted in Figure 1C. Electrons on ground state N_0_ are first pumped to excited state N_2_ through resonant energy transfer or direct self-absorption by a pulsed laser (*λ*_4_). After the pulse excitation is over, electrons still remain at N_2_ state for a short time due to the long luminescence lifetime of intermediate N_2_ state. Meanwhile, a donut-shaped laser *λ*_5_ matching the energy gap between N_2_ state and N_3_ is turned on all the time, which will consequently consume the electron population of N_2_ state and then result in elevated population of N_3_ state around the focal spot through the ESA process. Emission at *λ*_6_ from N_3_ state is much faster than that at *λ*_5_ from N_2_ state. Hence, in the region around the focal spot, electrons on N_3_ state are quickly depopulated to ground state N_0_, while in the central zone of the donut-shaped laser, most of the electrons still rest on N_2_ state. Finally, a short readout Gaussian pulse of *λ*_5_ laser is applied to generate the fluorescence signal from energy level N_3_ only in the finite central region. Therefore, a super-resolution image can be scanned through separated time–domain detection.

### Fluorescence Emission Difference

Fluorescence emission difference (FED) microscopy, also termed as switching laser mode (SLAM) microscopy, as a derivative method of STED, was independently proposed in theory and experiments by Xu Liu and Yves De Koninck in 2013 (Dehez et al., 2013; Kuang et al., 2013). In this design and the corresponding STED-like setup, two laser beams with Gaussian and donut intensity distributions are applied to stimulate bio-probes to generate fluorescence with solid and donut luminescence intensity distributions, respectively. Then two different images are subsequently collected based on the scanning imaging system of a conventional confocal microscope (Wen et al., 2020). The final super-resolution FED microscopic image is constructed using a subtractive intensity method of the above two images (Figure 1D). Some inevitably negative intensity values introduced by the subtraction process can be excluded by setting the value at zero to improve the imaging resolution. It should be noted that almost all the fluorescence probes are suitable for the super-resolution method, in which the optical resolution does not rely on a fluorescence depletion property under simultaneous excitation of two lasers but the emission saturation property of fluorescence probes. Lower laser power density to achieve maximum emission intensity will decrease the size of the dark spot in the emission donut pattern, thereby improving the optical resolution.

### Photon Avalanching Mechanism

Two-photon microscopy is a powerful tool for visualizing biological activities with deep-tissue imaging capability, but it is hard to resolve structures, such as cytoskeletons, with satisfactory spatial resolution (Helmchen and Denk, 2005). To address this issue, Artur Bednarkiewicz et al. proposed a novel concept that depends on using photon avalanching (PA) emission nanoparticles to explosively enhance the nonlinear relationship (*S*) between luminescence intensity and excitation power density (Bednarkiewicz et al., 2019). This technique utilizes the high *S* value (>50) to narrow the point spread function below 50 nm. Different from STED, this nonlinear laser scanning method for optical super-resolution exploits a single excitation laser, and the resolution increases according to the equation:

$$d=\;\frac{\lambda }{2\cdot \mathrm{NA}\cdot \sqrt{S}},$$ (2)

where *d* is the spatial resolution, *λ* is the excitation wavelength, NA is the numerical aperture of the oil immersion objective (typically NA = 1.4), and *S* is the degree of the nonlinear relationship between luminescence intensity and excitation power density. That is, to say, high nonlinearity will be beneficial for optical resolution. In a typical two-photon microscopic method, *S* value is about 2 (Ning et al., 2017). For lanthanide-doped nanoparticles, *S* can reach 5 (Chen et al., 2016). As to photon-avalanche nanoparticles, *S* can be adjusted to exceed 20, using which sub −70 nm spatial resolution can be realized (Lee et al., 2021).

## Performance Evaluation of Lanthanide-Doped Nanoparticles for Super-Resolution Imaging

Actually, one important role that makes the optical nanoscopy so powerful in bio-imaging and bio-detection is the great development of multifarious fluorescence probes that permit higher photostability, lower photon scattering and bio-toxicity, and so on. Until now, among various fluorescence probes, a series of nano-compositions doped with lanthanide ions, including Tm^3+^, Er^3+^, Pr^3+^, Dy^3+^, and Eu^3+^, have been used as fluorescence emitters for optical super-resolution imaging based on the STED-like setup. Different lanthanide ions exhibit varying abilities to break through the diffraction limit by disparate energy transfer channels and solution designs.

### Tm3^+^-Doped Nanocrystals for Super-Resolution Imaging

**FIGURE 2:**
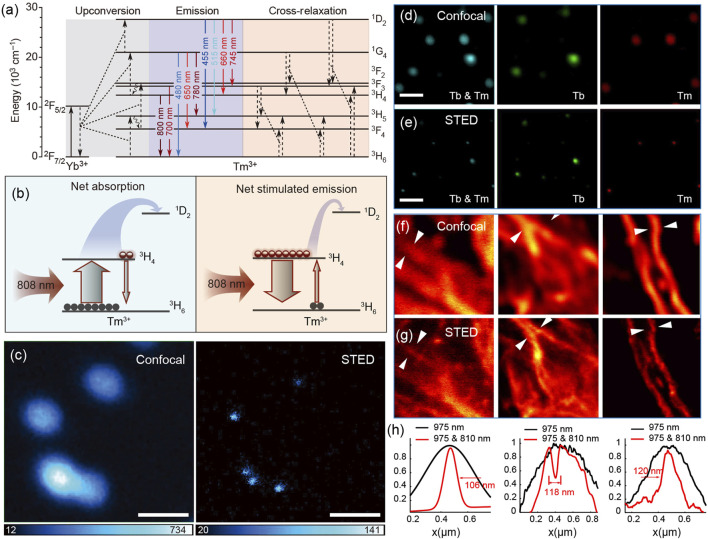
**(A)** Energy level diagram and energy transfer pathways of Yb^3+^/Tm^3+^ co-doped nanocrystals excited by a 980 nm laser. Solid arrows mean emissions; curved arrows mean energy transfer from Yb^3+^ to Tm^3+^ and typical cross-relaxation between Tm^3+^ ions. **(B)** Simplified diagrams describing net absorption **(left)** and net stimulated emission **(right)** between excited state ^3^H_4_ and ground state ^3^H_6_ of Tm^3+^ ions under a 980-nm laser excitation. **(C)** Optical imaging of 40 nm 8% Tm^3+^-doped nanocrystals based on confocal and STED microscopy, demonstrating the practicability of lanthanide-doped nanocrystals on super-resolution imaging. Adapted with permissions from the study by Liu et al. (2017) (scale bars, 500 nm). **(D)** Confocal and **(E)** super-resolution fluorescence images of NaGdF_4_:40%Yb^3+^,10% Tm^3+^@NaGdF_4_:15%Tb^3+^ nanocrystals with the emission wavelength at 455 and 547 nm **(left)**, 547 nm **(middle)**, and 455 nm **(right)**. **(F)** Confocal and **(G)** super-resolution fluorescence images of the cellular cytoskeleton protein desmin conjugated with Tm^3+^-doped nanocrystals. **(H)** Line profiles taken from **(F)** and **(G)** quantitatively indicate the improvement of optical resolution of STED nanoscopy. Adapted with permissions from the study by Zhan et al. (2017).

Activator Tm^3+^, often co-doped with sensitizer Yb^3+^, is a typically efficient blue-violet (475, 455, 360, 340 and 290 nm) light emitter under a continuous wave 980 nm laser excitation (Li et al., 2015; Wang et al., 2018). However, due to the much more intense cross-relaxation between Tm^3+^ ions than that between other activators (such as Er^3+^ and Ho^3+^), the optimal doping concentration of Tm^3+^ is relatively low, in the range of 0.2–1 mol% under excitation power density below 100 W/cm^2^ (Zhang et al., 2011). Thus, the upconversion luminescence intensity of Tm^3+^ is very weak because of the smaller number of activators (one of the main reasons). In 2013, Jin et al. found that 8% of Tm^3+^-doped nanocrystals at higher excitation irradiance (>10^6^ W/cm^2^) could generate strong upconversion luminescence at 800 nm that is about 70 times brighter than that in low Tm^3+^-doping nanocrystals (Zhao et al., 2013a). The mechanism investigation indicates that the saturated electron population of ^3^H_4_ state at high excitation power density induces an efficient energy transfer from the ^2^F_5/2_ state of Yb^3+^ to ^1^D_2_ and ^1^G_4_ states of Tm^3+^ ions. Subsequently, taking advantage of the high (8%mol) Tm^3+^-doped nano-composition, Jin reported a lanthanide-based STED microscopy for imaging single upconversion nanocrystals. In this study, 980 nm laser with a Gaussian intensity distribution acts as an excitation laser for illuminating the upconversion luminescence of Tm^3+^. At high excitation power density, almost all the electrons on ground state ^3^H_6_ are excited to state ^3^H_4_, resulting in a sharp population inversion between ^3^H_4_ and ^3^H_6_ states. At the same time, an 808-nm laser, matching only the ^3^H_4_ to ^3^H_6_ transition, stimulates disturbance on the electrons of the ^3^H_4_ state to produce stimulated emission at 808 nm, thus largely reducing the population of the ^3^H_4_ energy level and those of the higher states (^1^D_2_ and ^1^G_4_) that strongly depend on the population of ^3^H_4_ (Figures 2A andB). Combining the unique optical property of NaYF_4_:Yb^3+^/8%Tm^3+^ nanocrystals under two laser simultaneous excitation with an STED technique, Jin realized optical super-resolution imaging of a single nanocrystal with a saturation intensity (means the depletion laser power density that halves fluorescence intensity) as low as 0.19 MW/cm^2^ and spatial resolution of 28 nm, 1/36th of the excitation wavelength (Figure 2C).^26^ This work brings a very valuable application of lanthanide fluorescence probes on super-resolution imaging because the lanthanide nanocrystals possess excellent photostability compared with conventional organic dyes used in STED microscopy.

Almost at the same time, Zhan independently reported a similar stimulated emission phenomenon of high (10% mol) Tm^3+^-doped nanocrystals excited simultaneously by 975 and 810 nm lasers (Zhan et al., 2017). In his article, more detailed and in-depth researches on bio-applications were carried out. First, using NaYF_4_:Yb^3+^/Tm^3+^ nanocrystals (blue emission) together with NaGdF_4_:Yb^3+^/Tm^3+^@NaGdF_4_:Tb^3+^ nanocrystals ( green emission) that originates from the energy of ^1^I_6_ (Tm^3+^) through an energy migration–mediated upconversion (EMU) process, the two-color super-resolution imaging was exhilaratingly achieved (Figures 2D,E). Second, the super-resolution imaging of immunostained HeLa cells using lanthanide nanocrystals was first demonstrated, indicating the real practicability of lanthanide nanocrystals on STED microscopy (Figures 2F–H).

However, because of the electric dipole forbidden nature of the 4f–4f transitions and the shielding effect of the outer electronic orbit in trivalent lanthanide ions, the luminescence lifetimes of lanthanide-doped nanocrystals are typically long, ranging from tens of microseconds to milliseconds (Lu et al., 2013; Tan et al., 2020). However, in the STED microscopic scanning imaging system, longer luminescence lifetimes will inevitably reduce imaging scanning speed and lengthen the acquisition times of a super-resolution image, which limits the applications of the lanthanide-based STED technique in investigations of fast dynamic life processes and 3D volume super-resolution imaging. To address this challenge, Zhan et al. subtly designed high Yb^3+^-doped nanocrystals NaYF_4_@NaYbF_4_:10%Tm^3+^ to enhance upconversion luminescence by intensifying the local energy supply from Yb^3+^ to Tm^3+^ and meanwhile shorten the 455-nm emission lifetime from 34.32 to 7.45 µs due to the increased energy transfer speed between Yb^3+^ and Tm^3+^ (Peng et al., 2019). Compared to the earlier reported nanocrystals (NaYF_4_:18%Yb^3+^/10%Tm^3+^) that show long luminescence at 455 nm, the newly developed nanocrystals (NaYF_4_@NaYbF_4_:10%Tm^3+^) used for fast scanning super-resolution microscopy with a speed of 10 µs per pixel exhibit less emission streaking and higher spatial resolution. This work provides the potential for lanthanide-based STED microscopy to record dynamic vital activities.

By view of the abundant and ladder-like energy levels in trivalent thulium ions, the co-illumination of different stimulation wavelengths on low Tm^3+^-doped nanocrystals can induce the depleted fluorescence as well. In 2017, we also reported the quenched upconversion luminescence of NaYF_4_:Yb^3+^/Tm^3+^ nanocrystals under simultaneous excitations of 980 and 1,550 nm lasers (Zhang et al., 2017). The depletion ratio of emission around 455 nm could reach 90% at a depletion laser power density lower than 100 W/cm^2^. Such high-depletion efficiency at a low irradiation energy flux portends an underlying superior performance on super-resolution imaging. Great efforts were conducted to evaluate the possible depletion mechanism, including cross-relaxation, thermal effect, and stimulated emission. Last, we demonstrated that stimulated emission was responsible for the quenched fluorescence phenomenon at two-color fields. However, it should be noted that excitation sources with the wavelength around 1,500 nm are unamiable for bio-imaging and detection due to strong water absorption around the photon wavelength at 1,500 nm.

As for bio-applications, the emission at 455 nm of Tm^3+^ is not suitable for deep tissue super-resolution imaging because the luminescence intensity of four-photon upconversion (455 nm) will be seriously attenuated through deep tissues, and photon scattering of short wavelengths in deep tissues can result in reduced resolution as well (Hong et al., 2014). Therefore, in 2018, Jin et al. proposed near-infrared (800 nm) emission saturation nanoscopy for deep tissue super-resolution imaging by the FED technique (Chen et al., 2018). In the study, they first revealed that 4% Tm^3+^-doped upconversion nanocrystals showed lower power density to realize a similar spatial resolution as that for the reported 8% Tm^3+^-doped nanocrystals. For the sake of deep tissue super-resolution imaging, the near-infrared emission around 800 nm of Tm^3+^ through the two-photon process was used. Their results demonstrated sub-50 nm optical resolution through an 88-µm liver tissue slice. Due to the obvious different saturation thresholds of 800 and 740 nm emissions from Tm^3+^-doped nanocrystals, donut-shaped and Gaussian-shaped emissions were simultaneously generated to enhance the spatial resolution (40 nm) and imaging quality through a method of Fourier domain heterochromatic fusion derived from the FED method (Chen et al., 2021). Actually, this is far from the limit of resolution and penetration depth. Investigations of longer near-infrared emissive probes (especially emission wavelengths longer than 1,000 nm) on deeper tissue super-resolution imaging still need to be pushed in the future.

Although the 800-nm luminescence emission used in super-resolution imaging has been reported, the power density of the excitation laser was as high as 5.5 MW/cm^2^ (Chen et al., 2018). In 2021, Lee experimentally reported optical super-resolution imaging using photon-avalanche nanoparticles (NaY_0.92_Tm_0.08_F_4_@NaY_0.8_Gd_0.2_F_4_) under a very low power density (Lee et al., 2021). In this design, 11,064 nm wavelength as excitation laser and 8% Tm^3+^-doped nanoparticles as fluorescent probes were used to obtain effective photon-avalanche due to the weak ground state absorption and intense excited state absorption. The degree of nonlinearity *S* can be larger than 20 under a power density below 10 kW/cm^2^, which is two orders of magnitude lower than that used in most STED nanoscopies (Xu et al., 2021). According to Equation 2, sub-70 nm optical resolution was experimentally achieved under a power density of 7.6 kW/cm^2^. Besides the low power density used in this work, the super-resolution imaging system is based on a scanning confocal microscopic system, which is easier to implement than the STED system.

### Er^3+^-Doped Nanocrystals for Super-resolution Imaging

Apart from lanthanide Tm^3+^ for nanoscopic studies, Er^3+^ with typical green and red emissions can also be used in super-resolution imaging. As early as in 2015, Zhan et al. had already proposed the concept of lanthanide-doped nanocrystals used in STED nanoscopy (Wu et al., 2015). They developed a co-excitation system of 795 and 1,140 nm lasers for depleting the green emission of Er^3+^ions. Two processes, the ESA of the energy level ^2^H_11/2_ and ^4^S_3/2_ on 1,140 nm photon energy and energy transfer from the higher energy state (^4^G_11/2_) of Er^3+^ to Yb^3+^ions, synergistically contribute to the photo-induced depletion of green emissions. However, such a low depletion efficiency (maximum: 30%) cannot meet the practical requirements of super-resolution imaging in STED-like nanoscopy. Maybe, more efficient energy acceptors, such as organic dyes and quantum dots, will induce a higher fluorescence depletion performance.

Subsequently, to break through the annoying limit of depletion efficiency on imaging resolution, an FED technique was used for multiphoton super-resolution imaging based on red emission nanocrystals NaYF_4_:Nd^3+^/Yb^3+^/Er^3+^@NaYF_4_:Nd^3+^ with an 808-nm laser excitation. The spatial resolution in the design could reach 80 nm (Wu et al., 2017). Nonetheless, in FED imaging, two images at a solid and donut lasers excitation are scanned successively, which is time-consuming and suffers from sample jitters under high laser power density excitation. In view of this issue, core/multi-shell NaYF_4_:2%Er^3+^@NaYF_4_@NaYF_4_:20%Yb^3+^/2%Tm^3+^ nanocrystals with independent orthogonal excitation/emission luminescence at 808 and 940 nm laser excitation were developed and used as fluorescence probes in the two-detection-channel FED microscopy (Huang et al., 2018). Blue emissions of Tm^3+^ excited by a solid 940-nm laser and green emissions of Er^3+^ ions excited by a donut-shaped 808-nm laser could be collected synchronously, reducing the acquisition time in contrast to the conventional method of ordinal collection and ensuring the exactly identical position of the pixels in two images. After straightforward subtraction of the two images, a feature resolution of 54 nm was realized.

Er^3+^-doped nanocrystals, exhibiting intense emissions at both visible (540 and 654 nm) and near-infrared (1,525 nm) ranges, have been widely applied in diagnosis, therapy, and *in vivo* bio-imaging with high spatial resolution (Zhong et al., 2017; Wang et al., 2019; Zhang et al., 2019; Gao et al., 2019). However, an effective excitation/emission system to deplete the upconversion and downshifting luminescence intensity in Er^3+^-doped nanocrystals under two lasers irradiation has not been reported so far. For a better optical imaging resolution of *in vivo* STED nanoscopy, it is really desired to explore more feasible doping nanostructures of Er^3+^ ions.

### Other Lanthanide Ion–Doped Nanocrystals for Super-Resolution Imaging

**FIGURE 3:**
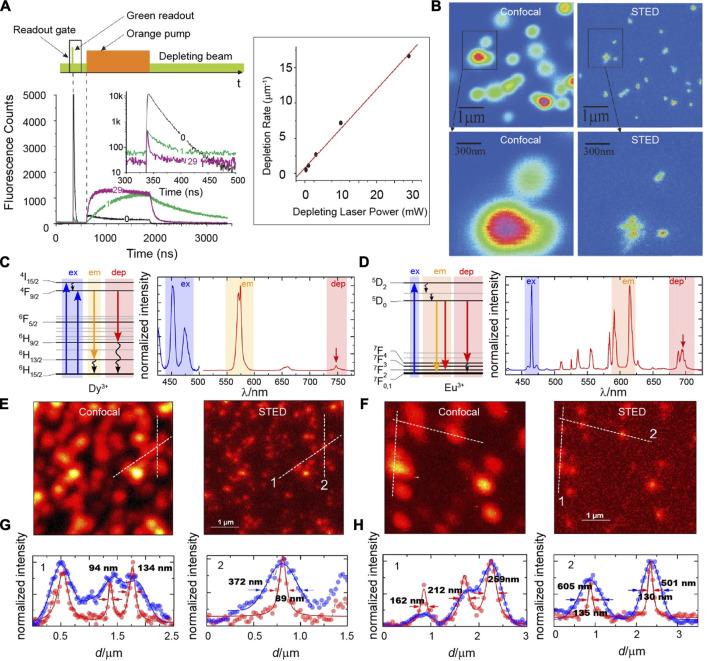
**(A)** Excitation sequence of pulsed and continuous wave lasers in time–domain **(upper)**, the decay curves of upconversion luminescence at different times **(lower)** and the depletion rates at different laser powers. **(B)** Confocal **(left)** and STED **(right)** images of *Pr*: YAG fluorescent nanoparticles. Adapted with permissions from the study by Kolesov et al. (2011). Simplified energy-level diagram **(left)**, excitation, and emission spectra **(right)** of **(C)** Dy^3+^ and **(D)** Eu^3+^ ions. Confocal **(left)** and STED **(right)** imaging of **(E)** NaYF_4_: Dy^3+^ and **(F)** NaYF_4_: Eu^3+^ nanocrystals at the same region. Intensity cross-section profiles of **(G)** NaYF_4_: Dy^3+^ and **(H)** NaYF_4_: Eu^3+^ nanocrystals of the dashed lines 1 and 2 in panels **(E)** and **(F)**. Adapted with permissions from the study by Stephan et al. (2019).

To the best of our knowledge, Pr^3+^ is the first lanthanide ion that is used in STED nanoscopy (Kolesov et al., 2011). In 2011, Kolesov described super-resolution imaging on ultraviolet emission nanocrystals (Pr: YAG) through an ESA mechanism (Figure 3A). Pr^3+^ had strong ground state absorption on 609 nm to populate its ^1^D_2_ state, while the excited state absorption from ^1^D_2_ to 4*f*5*d* (1) was only efficient at a wavelength of 532 nm rather than 609 nm. The population of ^1^D_2_ state in Pr^3+^ ions spatially located at the peripheral focus spot excited by a solid 609 nm pulsed laser could be consumed quickly through the ESA of the ^1^D_2_ energy level on a donut-shaped 532-nm continuous wave laser, resulting in a prior ultraviolet emission from 4*f*5*d* (1) of Pr^3+^ ions spatially around the focus spot. Because the luminescence lifetime of 4*f*5*d* (1) (18 ns) was much shorter than that of ^1^D_2_ (150–200 µs), the electron population of ^1^D_2_ state in the center of the focus spot was nearly unchanged after the 4*f*5*d* (1) state was absolutely depopulated. Finally, a short readout (20 ns) Gaussian pulse with a wavelength at 532 nm was used to generate ultraviolet emissions only at the dark spot of the donut laser. Based on the temporal control and STED equipment system, Roman Kolesov obtained sub-diffraction limit optical imaging on Pr: YAG nanocrystals with a spatial resolution of 50 nm that was restricted by the particle size (Figure 3B). The mechanism of ESA is very dependable and intriguing; however, it also requires a complicated optical system and precise synchronization control in temporal. A simpler optical system and rational design of fluorescence probes for super-resolution imaging based on the ESA mechanism still needs to be explored and developed.

The EMU process that mainly depends on the nanostructure NaGdF_4_:Yb^3+^/Tm^3+^@NaGdF_4_:X^3+^ is a promising energy transfer channel for the upconversion luminescence of those lanthanide activators without long-lived intermediary energy levels, such as Sm^3+^, Tb^3+^, Dy^3+^, and Eu^3+^ ions (Wang et al., 2011; Liang et al., 2019). Taking advantage of the mechanism and fluorescence depletion property of highly doped Tm^3+^ions, green emissions at 547 nm of Tb^3+^-doped EMU nanocrystals could be efficiently quenched and applied in STED super-resolution imaging (Zhan et al., 2017). This strategy can be readily extended to other lanthanide ions, including Eu^3+^, Dy^3+^, and Sm^3+^. However, no relevant further studies have been reported so far.

Recently, Stefan Krause had described a proof of the general lanthanide applicability on STED super-resolution imaging (Figures 3C–H) (Krause et al., 2019). A fluorescence intensity of 572 nm in NaYF_4_: Dy^3+^ nanocrystals directly excited by 449 nm, 452 nm, or 473 nm lasers could be exhausted by using a 748-nm laser co-excitation. A saturation intensity of 7.1 MW/cm^2^ for NaYF_4_: 3% Dy^3+^ nanocrystals was similar to what was measured for organic dyes, but it was obviously higher than those evaluated for Tm^3+^-based nanocrystals. As for NaYF_4_: Eu^3+^ nanocrystals, a 465-nm laser and a 695-nm laser were chosen to stimulate and deplete fluorescence around 600 nm, respectively. Similarly, the saturation intensity (3.3 MW/cm^2^) for NaYF_4_: 3% Eu^3+^ nanocrystals was so high that only a highly elevated depletion laser power density (222 MW/cm^2^) could guarantee an acceptable depletion efficiency and increased optical resolution.

To decrease the power density of the depletion laser, Liang et al. developed a novel lanthanide ion Nd^3+^-doped nanoparticle for STED nanoscopy. In the nanosystem, an 808-nm laser was used as an excitation source and a 1,064-nm laser was used as a depletion source to depopulate the excited state (^4^F_3/2_). Attributed to the long-lived metastable level (^4^F_3/2_, >50 µs) that is beneficial for population inversion, the saturation intensity was reduced to 19 kW/cm^2^, which enabled high-contrast deep-tissue imaging with about a 70-nm spatial resolution of subcellular structures. This work extends an important lanthanide ion in STED nanoscopy and will provide a great opportunity to achieve optical super-resolution using other lanthanide ions because the Nd^3+^ ion is a significant sensitizer to activate upconversion and downshifting luminescence of Er^3+^, Tm^3+^, Ho^3+^, and so on (Zhou et al., 2013b; Shen et al., 2013; Wang et al., 2013; Wang et al., 2014).

## Challenges and Outlook

Great efforts and improvements of lanthanide-doped nanocrystals in super-resolution imaging have been recently made and achieved. Until now, lanthanide ions including Tm^3+^, Er^3+^, Tb^3+^, Eu^3+^, Pr^3+^, and Dy^3+^ have been reported for potential super-resolution imaging on nanocrystals themselves and the cytoskeletons of HeLa cancer cells (Table 1). Despite these endeavors, the related development is still in its infancy. Several crucial challenges and issues still exist, involving the high power density of the depletion laser, a long luminescence lifetime of lanthanide probes, big particle size of lanthanide nanocrystals, short emission wavelength, and superior mechanism design, all of which need considerable explorations and investments from research fields of chemistry, materials science, and optics.

**TABLE 1:** Progress in the design and results of lanthanide-doped nanocrystals for super-resolution imaging.

Nanomaterials	Excitation laser/depletion or donut-shaped laser	Emission	Mechanism	Resolution	Laser power or power density for the resolution front column	Recent advances	Year
Pr: YAG	609 and 532 nm/532 nm	300–450 nm	ESA	50 nm	25 mW	First report to achieve super-resolution imaging by lanthanide luminescence materials based on an ingenious design of the ESA process.	Kolesov et al. (2011)
NaYF_4_: Yb^3+^/Er^3+^	795 nm/1,140 nm	525 nm, 550 nm	ESA	—	0.1 MW/cm^2^		Wu et al. (2015)
NaYF_4_: Yb^3+^/Tm^3+^, NaGdF_4_: Yb^3+^/Tm^3+^@NaGdF_4_: Tb^3+^	980 or 975 nm/808 or 810 nm	455 nm, 547 nm	STED	28/66 nm	9.75–17.7 MW/cm^2^	Developing a novel nano-component NaYF_4_: Yb^3+^/8–10%Tm^3+^ for one- or two-color STED nanoscopy	Zhan et al. (2017)
NaYF_4_: Yb^3+^/Tm^3+^	980 nm/1,550 nm	456 nm, 481 nm	STED	—	<0.1 kW/cm^2^		Zhang et al. (2017)
NaYF_4_: Yb^3+^/Nd^3+^/Er^3+^@ NaYF_4_: Nd^3+^	808 nm	650 nm	FED	80 nm	10 MW/cm^2^	Introducing Er^3+^ activated upconverting nanoparticles with non-photobleaching into FED microscopy	Wu et al. (2017)
NaYF_4_: Er^3+^@NaYF_4_@NaYF_4_: Yb^3+^/Tm^3+^	940 nm/808 nm	440–490 nm, 530–570 nm	FED	54 nm	1.78 MW/cm^2^		Huang et al. (2018)
NaYF_4_: Yb^3+^/Tm^3+^	980 nm	800 nm	FED	54 nm	5.5 MW/cm^2^	Proving the superior performance of 4% Tm^3+^-doped nanocrystals for super-resolution imaging	Chen et al. (2018)
NaYF_4_: Dy^3+^	449 nm, 452 nm, and 473/748 nm	572 nm	STED	89 nm	320 MW/cm^2^	Demonstrating a general approach using Dy^3+^ and Eu^3+^ single-doped nanocrystals for STED imaging applications	Krause et al. (2019)
NaYF_4_: Eu^3+^	465 nm/695 nm	590 nm, 615 nm	STED	130 nm	222 MW/cm^2^		
NaYF_4_@NaYF_4_: Yb^3+^/Tm^3+^	975 nm/810 nm	455 nm	STED	72 nm	—	Achieving a fast super-resolution imaging with 10 µs per pixel times	Peng et al. (2019)
NaYF_4_: 40%Yb^3+^/2%Tm^3+^	980 nm	800 nm/740 nm	FED	40 nm	2.75 MW/cm^2^	Only one excitation laser is employed to achieve high resolution with low laser power density	Chen et al. (2021)
NaY_0.92_Tm_0.08_F_4_@NaGdF_4_	1,064 nm	800 nm	PA mechanism	70 nm	7.6 kW/cm^2^	One excitation, low power density to achieve sub 70 nm resolution by photon avalanching	Lee et al. (2021)
NaGdF_4_: 1%Nd	808 nm/1,064 nm	850–900 nm	STED	20 nm	7.1 MW/cm^2^	A novel lanthanide ion used in STED with low saturation intensity	Liang et al. (2021)

**FIGURE 4:**
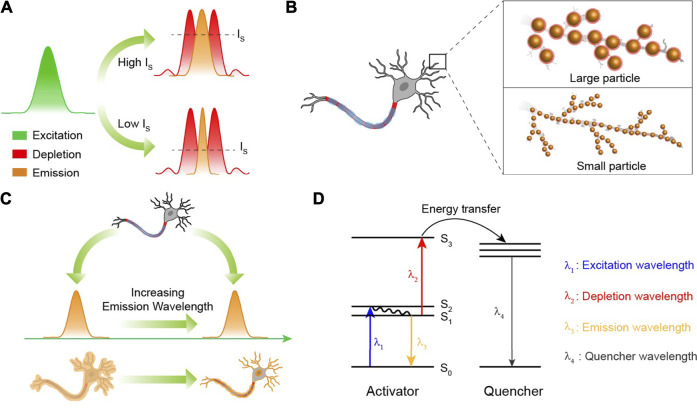
**(A)** The spatial region that fluoresces can be narrowed by reducing the saturation intensity (I_S_). Green, red, and yellow curves represent the areas of excitation, depletion, and emission, respectively. **(B)** The smaller nanoparticles that still possess a strong fluorescence intensity will ensure a higher spatial resolution than the larger ones. **(C)** In STED nanoscopy, the fluorescence probes with a longer emission wavelength will be favorable for *in vivo* super-resolution imaging due to their low photon scattering. **(D)** The excited state absorption mechanism for lanthanide ions in STED nanoscopy. *λ*_1_ is the wavelength of the excitation laser. *λ*_2_ is the wavelength of the depletion laser. *λ*_3_ is the emission wavelength. *λ*_4_ is the emission wavelength of the chosen quenchers. In the design, depletion laser, energy transfer from S_3_ of activator to quenchers and emissions from quenchers will continuously consume the population of S_1_state.

For a long time, organic compounds and fluorescent proteins were the commonly used fluorophores for STED nanoscopy that require fluorescent probes to be cycled many times between emission “on” and “off” states. Therefore, the photostablity of fluorescent probes is very important. Since lanthanide-doped nanocrystals show excellent photostability without photobleaching (Wang and Liu, 2009), they have great advantages on micro-imaging that usually needs high laser power density. However, this does not mean that the high power density is tolerable because there exists an underlying over-heating effect on biological specimens when a required spatial resolution only occurring at a high power density of the depletion laser is required. The current power density of the depletion laser for satisfying the spatial resolution is as high as 10 MW/cm^2^ (Table 1). A significant approach to decrease the demanded power density is to elevate the depletion efficiency, so as to lower the energy flux for an equivalent resolution. According to Equation 1, it is obvious that (1+IIS) is the inverse of the depletion ratio, indicating that the lower the $I_{S}$ value, the higher the depletion efficiency and the better the spatial resolution (Figure 4A). Therefore, developing novel fluorescence probes and energy transfer mechanisms with lower saturation intensity will alleviate the over-heating effect caused by the depletion laser, stabilizing the fluorescence intensity and boosting the optical resolution in STED nanoscopy. Although the reported $I_{S}$ value (0.19 MW/cm^2^) of Tm^3+^-doped nanocrystals is seemingly lower than that of commonly used organic dyes (Liu et al., 2017), the practical power density of the depletion laser for a benign spatial resolution is usually as high as 10 MW/cm^2^. There is still a large promotion potential to reduce the saturation intensity.

As is well known, STED nanoscopy is a point-by-point scanning imaging process. Hence, the imaging rates of STED nanoscopy usually depend on the pixel dwell times. Nevertheless, due to the shielding of the outer orbit in trivalent lanthanide ions, the luminescence lifetimes of lanthanide ions are uniquely long (>50 µs) (Zhao et al., 2013b; Lu et al., 2013; Wang et al., 2016), going against a quick frame capture. The imaging acquisition time is necessarily shortened when it comes to the dynamic imaging of certain biological activities. Therefore, effective approaches to greatly shorten the luminescence lifetime to a sub-microsecond level, and in the meantime reserve or enhance the emission intensity, is mightily desired to ensure brief pixel dwell times. Several methods including elevating the doping concentration of sensitizer Yb^3+^ and coupling gap plasmonic cavity are proposed (Su et al., 2017; Feng et al., 2019). So far, ultrabright spontaneous emission of Er^3+^ with ultrashort luminescence lifetimes (1.4 µs) has been realized through a finely designed nanocavity, where the nanostructures should be elaborately and specifically fabricated, leading to an inevitable obstacle for the freewheeling targeting distribution of the luminescence center on bio-specimens (Wu et al., 2019; Xu et al., 2021).

Imaginably, the size distribution of nanoparticles at some point will become a limiting factor because the spatial resolution of the present STED technique can reach below 30 nm (Gottfert et al., 2013; Kasper et al., 2010), indicating that a large nanoparticle (>30 nm) would restrain the improvement of the spatial resolution (Figure 4B). And for a better optical resolution, the size should be as small as possible, while fluorescence signals are still kept strong enough to be detected for super-resolution imaging. It is well known that nanocrystals based on the Gd^3+^ host exhibit ultrasmall particle sizes (<5 nm), yet accompanied by significantly quenched fluorescence intensity as well (Wong et al., 2011; Chen et al., 2019). Some efforts have been made to develop lanthanide-doped nanoparticles with bright upconversion luminescence at a single particle level. In 2018, NaYF_4_@NaYb_0.92_F_4_:Er_0.08_@NaYF_4_ nanoparticles exhibiting bright luminescence (upconversion quantum yield: 5.03 ± 0.60%) were reported by Steve Chu (Liu et al., 2018). Besides, an enhanced resolution (294.4 nm) of single nanoparticle (NaYF_4_: 60%Yb, 8%Er_0.08_@NaYF_4_) imaging was achieved due to its bright upconversion luminescence (Ma et al., 2020). However, a size distribution of more than 29 nm for these nanoparticles is unsatisfactory for optical super-resolution because a spatial resolution below 30 nm has been achieved through STED nanoscopy (Xu et al., 2021). It is still a great challenge to develop a general synthetic method or nano-architectural method to achieve bright fluorescence intensity from ultrasmall lanthanide-doped nanocrystals with a particle size less than 5 nm (Willets, 2013; Liu et al., 2021). In 2021, Gu et al. prepared NaGdF_4_: 1%Nd nanoparticles with 6.68 ± 0.8 nm size distribution to achieve STED super-resolution imaging (Liang et al., 2021), which is a considerable improvement in the particle size used in STED nanoscopy, and more efficient luminescence emission with smaller particle size of various lanthanide ion–doped nanoparticles is required in the future.

Here, we introduced and concluded the design and potential application of lanthanide-doped nanoparticles for optical super-resolution imaging. Present works mainly focus on the imaging of a single lanthanide-doped nanoparticle. The super-resolution imaging of cytoskeletons and intracellular microtubule structures based on lanthanide ions has been realized (Zhan et al., 2017; Liang et al., 2021). However, as can be seen, the bio-applications of super-resolution imaging based on lanthanide-doped nanoparticles developed slowly. One great challenge is that the conjugated efficiency of lanthanide-doped nanoparticles labeling the substructures of cells is low. Presently, conjugating these nanoparticles to subcellular structures by antibodies is a popular method.

Recently, scientists have demonstrated that fluorescence probes with second near-infrared (NIR II, 1,000 nm–1700 nm) emissions show superior tissue penetration and better imaging resolution because of reduced photon scattering and biological absorption at this wavelength region (Hong et al., 2014; Ding et al., 2018; Li et al., 2020; Zhao et al., 2021). The current emission wavelengths of lanthanide-doped nanocrystals used in STED-like nanoscopy are usually located at the visible region, which is not suitable for *in vivo* super-resolution imaging at a relatively deeper tissue. In 2018, an 800-nm emission from Tm^3+^-doped nanocrystals was used in STED-like nanoscopy to achieve super-resolution imaging through a 92-µm brain tissue slice, where only 11.3% of the fluorescence intensity at 455 nm was left due to the strong photon attenuation (Chen et al., 2018). So, developing a novel excitation system and lanthanide-doped nanocrystals with NIR II emissions that satisfy the requirement of *in vivo* super-resolution imaging beneath a deep tissue is also a significant research field (Figure 4C). It should be noted that in conversional optical and confocal microscopy, the imaging resolution principally relies on the emission wavelengths of fluorescence probes, while in STED-like nanoscopy, it is entirely different. According to Equation. 1, the spatial resolution is dependent on the wavelengths of depletion lasers rather than the emission wavelengths of fluorescence probes. So, prolonging the luminescence wavelength will not lead to the natural decrease of the imaging resolution.

FED microscopy, as a derivative design of the STED technique, breaks the limit that lanthanide ions applied in popular STED nanoscopy must have an efficient behavior of stimulated emission, meaning that almost all the fluorescent lanthanide ions can be used in FED microscopy. However, different from STED nanoscopy, the theoretical optical resolution in FED microscopy is intrinsically related to the emission wavelengths of lanthanide ions, resulting in increased difficulty in distinguishing adjacent lanthanide ions. Hence, a more complete data processing model is desired to promote the theoretical optical resolution of FED microscopy.

Novel solutions to achieve the efficient fluorescence depletion of targeted energy levels with lower $I_{S}$ values are required as well. Super-resolution imaging based on the ESA mechanism is very promising due to the low threshold (<1 kW/cm^2^) of the ESA process (Sun et al., 2019; Auzel, 2004; Yan et al., 2018). Combining lanthanide ions with potential acceptors with high-absorption cross-section under two laser excitation, the electron population of high energy states in lanthanide ions could be greatly exhausted in principle, thus reducing the electron population of the low energy level that occurs in the ESA process (Figure 4D). Besides, super-resolution imaging of lanthanide-doped nanoparticles using structured illumination microscopy (SIM) has also been reported recently. This combination leads to a new modality microscopy with a resolution below 130 nm, 1/7th of the excitation wavelength (976 nm) (Liu et al., 2020). Meanwhile, Hu developed a multiphoton upconversion time-encoded structured illumination microscopic (MUTE-SIM) method to obtain ultrafast super-resolution multiphoton imaging with a scanning rate of 50 MHz (Hu et al., 2020). However, the present structured illumination microscopy using lanthanide-doped nanoparticles as fluorescent probes still cannot achieve an optical super-resolution below 50 nm. Further studies to drive these super-resolution applications on *in vivo* imaging still need to be pushed. Besides SIM and its derived techniques, other methods, such as stochastic optical reconstruction microscopy (STORM), can also realize significant optical super-resolution imaging (Wang et al., 2019). However, works of STORM based on lanthanide-doped nanoparticles have not been reported so far. This is mainly due to the fact that fluorescent probes applied in STORM must be able to be stochastically switched between a fluorescent and a nonfluorescent status at a single-molecule level, which is attributed to fluorophore blinking in the presence of excitation light (Bates et al., 2005; Huang et al., 2008), while lanthanide-doped nanoparticles exhibit excellent photostability and non-photobleaching (Wang et al., 2019; Pei et al., 2021), resulting in a big barrier for using lanthanide-doped nanoparticles in STORM. In addition to this, STORM establishes an on and off state at the single-molecule level so that the theoretical optical resolution of STORM may be below 1 nm (Bates et al., 2008; Li and Vaughan, 2018), which is much less than the particle size of the lanthanide-doped nanoparticles with bright luminescence (Zhang et al., 2020; Zhao et al., 2020).

In summary, we introduced and analyzed a recent process of lanthanide-doped nanocrystals on super-resolution imaging. Exciting works in the multidisciplinary field including chemistry, nanomaterials, and optics have already established the scientific fundamentals and design solutions for higher spatial resolution and more practical applications. Under the inspiring achievements, great efforts on developing superior emission conditions of lanthanide ions that satisfy the demand of super-resolution imaging should be made in the future.
